# Research Upregulation of CD23 (FcεRII) Expression in Human Airway Smooth Muscle Cells (huASMC) in Response to IL-4, GM-CSF, and IL-4/GM-CSF

**DOI:** 10.1186/1476-7961-3-6

**Published:** 2005-05-20

**Authors:** Joseph T Belleau, Radha K Gandhi, Holly M McPherson, D Betty Lew

**Affiliations:** 1 Department of Pediatrics, Children's Foundation Research Center at the Le Bonheur Children's Medical Center, University of Tennessee Health Science Center, 50 North Dunlap Street, Rm401, WPT, Memphis, TN 38103, USA

## Abstract

**Background:**

Airway smooth muscle cells play a key role in remodeling that contributes to airway hyperreactivity. Airway smooth muscle remodeling includes hypertrophy and hyperplasia. It has been previously shown that the expression of CD23 on ASMC in rabbits can be induced by the IgE component of the atopic serum. We examined if other components of atopic serum are capable of inducing CD23 expression independent of IgE.

**Methods:**

Serum starved huASMC were stimulated with either IL-4, GM-CSF, IL-13, IL-5, PGD2, LTD4, tryptase or a combination of IL-4, IL-5, IL-13 each with GM-CSF for a period of 24 h. CD23 expression was analyzed by flow cytometry, western blot, and indirect immunofluorescence.

**Results:**

The CD23 protein expression was upregulated in huASMC in response to IL-4, GM-CSF, and IL-4/GM-CSF. The percentage of cells with increased fluorescence intensity above the control was 25.1 ± 4.2% (IL-4), 15.6 ± 2.7% (GM-CSF) and 32.9 ± 13.9% (IL-4/GMCSF combination)(n = 3). The protein content of IL-4/GMCSF stimulated cells was significantly elevated. Expression of CD23 in response to IL-4, GM-CSF, IL-4/GM-CSF was accompanied by changes in cell morphology including depolymerization of isoactin fibers, cell spreading, and membrane ruffling. Western blot revealed abundant expression of the IL-4Rα and a low level expression of IL-2Rγc in huASMC. Stimulation with IL-4 resulted in the phosphorylation of STAT-6 and an increase in the expression of the IL-2Rγc.

**Conclusion:**

CD23 on huASMC is upregulated by IL-4, GM-CSF, and IL-4/GM-CSF. The expression of CD23 is accompanied by an increase in cell volume and an increase in protein content per cell, suggesting hypertrophy. Upregulation of CD23 by IL-4/GM-CSF results in phenotypic changes in huASMC that could play a role in cell migration or a change in the synthetic function of the cells. Upregulation of CD23 in huASMC by IL-4 and GM-CSF can contribute to changes in huASMC and may provide an avenue for new therapeutic options in asthma targeting ASMC.

## Background

Chronic inflammation and airway smooth muscle dysfunction are consistent features of asthma responsible for disease progression and airway remodeling [1]. The increase in bronchial smooth muscle, both hypertrophy [2] and hyperplasia [3], plays a critical role in the development of airway hyperreactivity (AHR), the hallmark of asthma. Airway smooth muscle cells (ASMC) may also play a secretory or immunomodulatory role by producing pro-inflammatory cytokines, chemokines, polypeptide growth factors, extracellular matrix proteins, cell adhesion receptors, and co-stimulatory molecules, which perpetuate submucosal inflammation [4,5]. These mediators may act on the ASM itself in an autocrine manner as well to further contribute to the asthma phenotype [6]. Therefore, smooth muscle itself may be capable of initiating and maintaining airway inflammation. Also, ASMC have been shown to undergo cell migration, which could contribute to airway remodeling [7]. Thus, regulation of airway smooth muscle hypertrophy and migration may be a new target for treatment of asthma [7,8].

It is well known that IgE plays a critical role in the pathogenesis of asthma in the early and late phases by interacting with its two receptors, the high affinity receptor (FcεRI) and the low affinity receptor (FcεRII) [9]. IgE plays a key role in bronchial hyperresponsiveness and smooth muscle hyperreactivity [8]. Crosslinking of the high affinity IgE receptor (FcεRI) on mast cells leads to cellular degranulation and the release of various proinflammatory mediators and cytokines contributing to bronchoconstriction. The low affinity IgE receptor (CD23) (FcεRII) has been identified on B cells, monocytes, follicular dendritic cells, Langerhan's cells, eosinophils, and platelets [10]. Upregulation of the CD23 receptor is thought to increase allergic responses in the bronchial mucosa through the enhancement of antigen uptake and presentation [8]. The receptor has two isoforms that differ only in their cytoplasmic domains [11]. CD23a is constitutively expressed on B cells and is associated with endocytosis of IgE coated particles, and CD23b is induced by IL-4 and is also found on non- B cells such as T cells, Langerhan's cells, monocytes, macrophages, platelets, and eosinophils [12,13]. IL-4 causes CD23 induction on B cells through CD40 [12]. CD23b mediates phagocytosis of soluble IgE complexes. An autocatalytic process involving cleavage of membrane bound CD23 by a matrix metalloprotease yields a series of soluble elements (sCD23) which increase IgE production via the CD21 receptor on B cells [13,14].

The CD23 receptor has been shown to be upregulated on monocytes and alveolar macrophages in a T helper cell type 2 (TH2) environment and may contribute to chronic inflammation in asthma through this mechanism [15]. It has been shown that IL-4 and GM-CSF induce CD23 expression on monocytes, and GM-CSF primes monocytes for cellular activation and secretion of IL-1 upon subsequent exposure to IgE-containing immune complexes [8]. CD23 is also involved in antigen presentation to B cells as well as cellular interactions between B and T cells [12].

In previous studies, Hakonarson et al. [16] demonstrated the expression of CD23 (FcεRII) messages and a low level of the protein in airway smooth muscle cells. In two patients who died of status asthmaticus, CD23 expression was also markedly upregulated on ASMC. The CD23 expression was inducible with human atopic sera or IgE immune complexes in naïve (control) ASMC, and this upregulation was blocked when pretreated with anti-CD23 blocking antibody. The authors concluded that IgE coupled activation of CD23 contributes largely to its upregulation [5,14]. In a corresponding experiment with rabbit ASMC subjected to control and atopic serum, they were able to demonstrate, through Western blot analysis, a markedly enhanced expression of CD23 in the ASMC sensitized with atopic serum or IgE immune complexes. They were able to achieve significant inhibition of upregulation by pretreatment with anti-CD23 mAb. They hypothesized that IgE was responsible for the upregulation of the low affinity IgE receptor [17]. Hakonarson, et al. have also demonstrated that ASMC in vitro exposed to human atopic sera results in an initial increase in TH2 cytokines including IL-5 and GM-CSF followed hours later by production of IL-1 and TH1 cytokines [18,19]. Recently, phase I trials have been completed on IDEC-152, an IgG1 anti-CD23 antibody, for patients with mild to moderate persistent asthma. The drug was well tolerated by participants, and a dose-dependent decrease in mean IgE values was reported [20].

T helper cell type 2 (TH2) mediated inflammatory cytokines, such as IL-4, IL-13, and IL-5, as well as other enzymes and chemokines are active in the asthmatic patient. GM-CSF has been shown to be involved in asthma pathogenesis and in vivo can induce TH2 differentiation independent of IL-4 [21]. Tryptase and prostagladin D2 (PGD2) were chosen as major mast cell mediators, and more recently the PGD2 receptor gene (PTGDR) has been shown to be an asthma susceptibility gene [22-24]. It is possible that many factors are responsible for the upregulation of the low affinity IgE receptor in addition to and independent of IgE. The purpose of this study was to identify specific mediators released in the asthmatic patient that are responsible for the upregulation of CD23 on human airway smooth muscle cells independent of IgE.

## Methods

### Cell culture and flow cytometry

**Figure 1:**
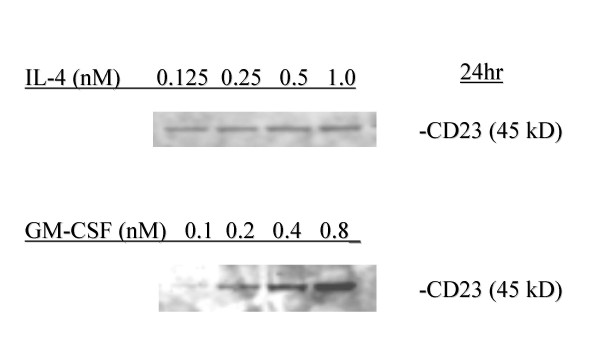
**Upregulation of CD23 by IL-4 and GM-CSF**. Dose-ranging studies were performed to determine the optimum concentrations of IL-4 and GM-CSF. Alpha-smooth muscle isoactin positive Human ASMC (Clonetics) in T-75 flasks were starved for 24 h in 0.1% FBS containing medium M199. The cells were then stimulated with BSA (1 μg/ml), IL-4 (0.125. 0.25, 0.5, or 1.0 nM) or GM-CSF (0.1, 0.2, 0.4, or 0.8 nM) for 24 h. The cell lysates in RIPA buffer were subjected to western blot analysis for CD23. Mouse anti-human CD23 monoclonal antibody (clone M-L233, BD Biosciences, 1 μg/5 ml) was used as the primary antibody and anti-mouse horseradish peroxidase linked antibody as the secondary antibody (Amersham). The immunoreactive protein bands were detected by enhanced chemiluminescence light (ECL) (Amersham).

Alpha-smooth muscle isoactin positive Human ASMC (Cambrex, Walkersville, MD) in T-75 flasks were starved for 24 hours in 0.1% (vol/vol) fetal bovine serum (FBS) containing medium M199 (Cellgro, Herndon, VA) supplemented with 1% (vol/vol) antibiotic/antimycotic solution (Sigma Chemical Co., St Louis, MO). The cells were then stimulated with either vehicle (bovine serum albumin, BSA, vehicle for cytokines (1 mg/ml), in M199; ethanol (EtOH), vehicle for LTD4 (6% final concentration) and PGD2 (0.001–0.01% final concentration); M199, vehicle for tryptase), an individual mediator, or a mediator in combination with GM-CSF at their optimum concentrations for 24 hours. The doses of cytokines used were up to four time ED50 including: IL-4 (0.04–1 nM), GM-CSF (0.07–0.8 nM), IL-13 (0.4 nM), IL-5 (0.01–0.07 nM), IL-13 (0.3–2.2 nM), PGD2 (1–10 μM), LTD4 (1–10 μM), tryptase (30 nM, a concentration sufficient to induce ASMC proliferation) (Sigma). Dose ranging studies were performed to determine the optimum concentration of IL-4 and GM-CSF on the expression of CD23, and the doses chosen were IL-4 (0.5 nM) and GM-CSF (0.4 nM) (Figure 1). All cytokines were obtained from R & D Systems Inc. Minneapolis, MN except GM-CSF which was obtained from Sigma. The cells were then harvested with a soft rubber edged scraper, centrifuged for 5 minutes at 1000 rpm (200 g), washed and resuspended in 1% BSA in phosphate buffered saline (PBS) and fixed with 70% ETOH. After washing twice more, the cells were resuspended in 1% BSA in PBS. Finally, they were filtered through a 40 μm nylon mesh to obtain single cell suspension and stained with (20 μl) of PE (phycoerythrin)-CD23 (EBVCS-5, BD Biosciences, San Jose, CA) or PE-mouse IgG_1 _for 15 minutes in the dark to facilitate staining for flow cytometry.

### Protein analysis

A commercially available bicinchoninic acid (BCA) kit (Pierce, Rockford, IL) was used for protein analysis according to the manufacturer's instructions. The optical densities were read using a Bio-Kinetics EL-312 Microplate reader.

### Indirect immunofluorescence

Indirect immunofluorescence stainings were performed with anti-smooth muscle-α isoactin antibody (Sigma) and anti-human CD23 antibody (M-L233, 1 μg/ml, BD Biosciences), which are specific monoclonal antibodies and either a FITC or TRITC fluorochrome, conjugated second antibody. Fixed huASMC were incubated with the above antibodies diluted in PBS with 3% BSA for 60 min at room temperature. The cells were then washed three times with PBS for 10 minutes for each wash. Non-specific binding was blocked by incubating cells with 3% BSA in PBS for 60 minutes. The blocking solution was then removed and cells were incubated with FITC- or TRITC- fluorochrome conjugated antibody for 45 minutes in the dark to facilitate staining. Cells were then washed with PBS three times. Finally, one drop of Fluoromount-G (Southern Biotechnology Inc., Birmingham, AL) was added.

### Western blot

Standard Western blot analyses were performed to detect anti-STAT6 (1:500, Calbiochem, San Diego, CA) polyclonal rabbit, anti-p-STAT-6 (1:500, Calbiochem) polyclonal rabbit. Human ASMC lysates in radio-immunoprecipitation assay (RIPA) buffer were transferred onto Hybond-ECL nitrocellulose membranes and were immunoblotted with monoclonal anti-human CD23 (1:500 dilution, clone M-L233, 1 μg/5 ml, BD Biosciences), polyclonal anti-IL-4Rα (1:500 dilution, Santa Cruz), monoclonal anti-IL-2Rγc (1:250 dilution, R&D Systems, Inc.). The nitrocellulose membranes were incubated with a 1:1,000 dilution of anti-rabbit or anti-mouse horseradish peroxidase linked whole antibody (Amersham, Piscataway, NJ) in PBS-T for 1 hour at room temperature. Paxillin monoclonal antibody (1:500 dilution, Transduction Laboratories) was used as a positive isotype control for CD23, and fibronectin polyclonal antibody (1:250, Sigma) was used as a positive control for the remaining antibodies. The immunoreactive protein bands were detected by enhanced chemiluminescence light (ECL) (Amersham).

### Statistical analysis

Data were analyzed with Prism 4 software (GraphPad, San Diego, CA). One-way analysis of variance (ANOVA) was used. Results are expressed as mean ± SEM. A P value less than 0.05 was considered statistically significant.

## Results

### CD23 protein expression is upregulated in huASMC by IL-4, GM-CSF, or IL-4/GM-CSF

**Figure 2:**
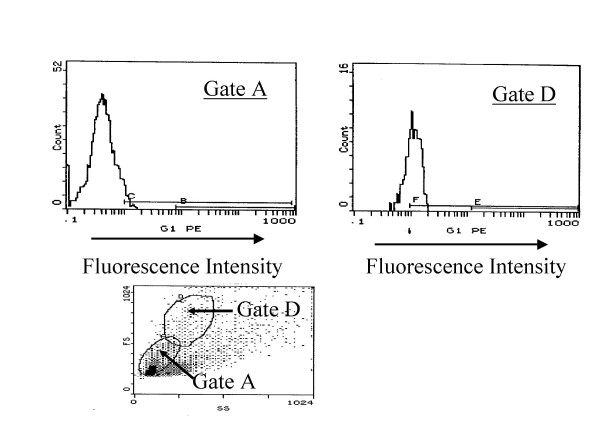
**Upregulation of CD23 by IL-4, GM-CSF and IL-4/GM-CSF**. Alpha-smooth muscle isoactin positive Human ASMC (Clonetics) in T-75 flasks were starved for 24 h in 0.1% FBS containing medium M199. The cells were then stimulated with IL-4 (0.5 nM)/GM-CSF (0.4 nM) for 24 h. The FACS analysis showed that the smaller cells passed through Gate A and larger cells passed through Gate D. Background noise was eliminated using the BSA-stimulated control cells that were labeled with PE-anti-CD23 (EBVCS), represented by C in Gate A, and F in Gate D. The FACS results of a representative experiment showed 66% of the larger cells (Gate D) had an increase in cell expression of CD23 when compared to the controls.

Previous studies have shown that IgE immune complexes in atopic serum caused an increase in CD23 expression in ASMC [16]. To determine if other humoral factors in atopic serum effect CD23 expression in human ASMC, we have tested the effect of the relevant cytokines, arachidonic acid metabolites, and the mast cell enzyme tryptase. Flow cytometry was performed to evaluate differences in cell populations after stimulation of the huASMC for 24 hours with either individual mediators IL-4 (0.5 nM), GM-CSF (0.4 nM), IL-13 (0.4 nM), IL-5 (0.4 nM), PGD2 (10 μM), LTD4 (10 μM), tryptase (30 nM) or a combination of IL-4, IL-5, and IL-13 each with GM-CSF. Within the huASMC stimulated by IL-4, GM-CSF or the combination of IL-4/GM-CSF, two populations of cells were detected distinguishable by cell size. While the smaller cells did not show a significant expression of CD23, many of the larger cells showed increased expression of CD23. In the example in Figure 2, 66% of the larger cells (gate D) showed an increase in cell expression of CD23 when compared to the controls. As stated previously, the functions of ASMC are heterogeneous including proliferation and synthesis. Previous studies have shown, on flow cytometry of ASMC stimulated in vitro with IL-1β and TNF-α, only 20–60% of ASMC produce GM-CSF. The ASMC producing GM-CSF include some which also have increased proliferative properties. This suggests that considerable heterogeneity exists in the phenotypic expression of the ASMC in culture [25].

In addition to the combination of IL-4/GM-CSF inducing increased expression of CD23, both IL-4 and GM-CSF alone independently increased the expression of CD23 in huASMC. The percentage of cells with increased fluorescence intensity above the control was 25.1 ± 4.2% (IL-4), 15.6 ± 2.7% (GM-CSF) and 32.9 ± 13.9% (IL-4/GM-CSF combination). On the other hand, IL-5, IL-13, cysteinyl leukotrienes, and tryptase did not induce CD23 expression (Table 1).

**Table 1:** Il-4, GM-CSF, and IL-4/GM-CSF Increase CD23 Expression on ASMC (n = 3)

Cytokine (24 hours)	#Cells in Gate D (n = 3)	%Cells with Increased CD23 Expression Above the Control
BSA (1 μg/ml)	1,343 ± 122	0
IL-4 (0.5 nM)	1,413 ± 197	25.1 ± 4.2*
GM-CSF (0.4 nM)	1,346 ± 243	15.6 ± 2.7*
IL-4/GM-CSF (0.4/0.5 nM)	1,324 203	32.9 ± 13.9*
IL-5 (0.4 nM)	1,130 ± 251	0
IL-13 (0.4 nM)	1,316 ± 269	0
PGD2 (1 μM)	1,521 ± 123	0
PGD2 (10 μM)	1,159 ± 204	0
LTD4 (10 μM)	2,037 ± 375	0
Ethanol (6% vol/vol)	2,507 ± 200	0
Tryptase (10 μM)	2,385 ne ± 405	0

Alpha-smooth muscle isoactin positive Human ASMC (Clonetics) in T-75 flasks were starved for 24 h in 0.1% FBS containing medium M199. The cells were then stimulated with BSA (1 μg/ml), IL-4 (0.5 nM), GM-CSF (0.4 nM), IL-4 (0.5 nM)/GM-CSF (0.4 nM), IL-13 (0.4 nM), IL-5 (0.4 nM), PGD2 (10 μM), LTD4 (10 μM), tryptase (30 nM) for 24 h. Results are mean ± SEM of the percentage of cells with an increased fluorescence intensity above the control (n = 3). Control values: BSA-stimulated, PE-anti-CD23 labeled, 10.9 ± 1.4 %; BSA-stimulated, PE-mouse IgG_1_, non-immune, 0.5 ± 0.1 % (n = 3). *denotes significant increase in CD23 expression above the BSA control value.

### Expression of CD23 in response to IL-4, GM-CSF, IL-4/GM-CSF is accompanied by changes in huASMC morphology

**Figure 3:**
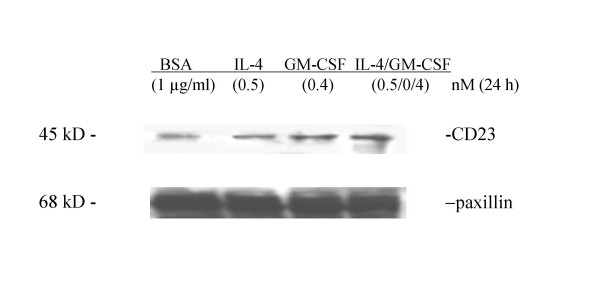
**Western blot analysis of CD23 after stimulation of IL-4, GM-CSF, IL-4/GM-CSF**. Alpha-smooth muscle isoactin positive huASMC (Clonetics) in T-75 flasks were starved for 24 h in 0.1% FBS containing medium M199. The cells were then stimulated with BSA (1 μg/ml) (vehicle control), IL-4 (0.5 nM), GM-CSF (0.4 nM), or IL-4/GM-CSF (0.5 nM/0.4 nM) for 24 h. The cell lysates in RIPA buffer were subjected to western blot analysis for CD23. Mouse anti-human CD23 monoclonal antibody (clone M-L233, BD Biosciences, 1 μg/5 ml) was used as the primary antibody and anti-mouse horseradish peroxidase linked antibody as the secondary antibody (Amersham). The immunoreactive protein bands were detected by enhanced chemiluminescence light (ECL) (Amersham). Paxillin mouse monoclonal IgG_1 _(Transduction Laboratories) was used as an irrelevant isotype control.

**Figure 4:**
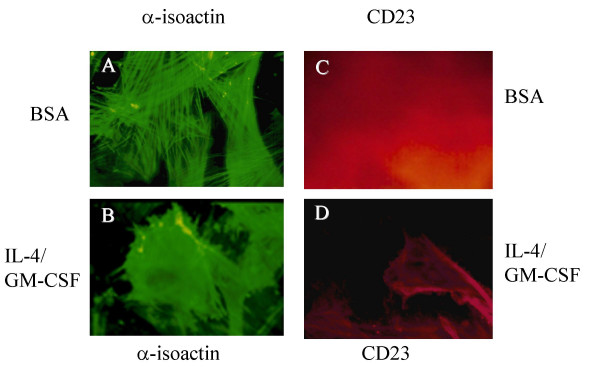
**Expression of CD23 in response to IL-4/GM-CSF is accompanied by changes in huASMC morphology**. Alpha-smooth muscle isoactin positive huASMC (Clonetics) in T-75 flasks were starved for 24 h in 0.1% FBS containing medium M199. The cells were then stimulated with either BSA or IL-4 (0.5 nM)/GM-CSF (0.4 nM) for 24 h, and stained with either anti-smooth muscle-isoactin (A & B) or anti-CD23 antibody (C & D). Those cells stimulated with the combination of IL-4/GM-CSF demonstrated CD23 expression (D) and changes in cell morphology including depolymerization of isoactin fibers, cell spreading, and membrane ruffling (B). Cells stimulated with BSA (vehicle for IL-4/GM-CSF) alone did not increase the expression of CD23 (C) nor changes in phenotype (A). These findings were confirmed by three independent observers.

Western blot analysis of huASMC stimulated with IL-4, GM-CSF, or Il-4/GM-CSF for 24 h showed an increase in CD23 expression compared to BSA vehicle control (Figure 3). Indirect immunofluorescence was used also to identify any morphological changes associated with the cytokine stimulation and upregulation of CD23 (Figure 4A–D). Those cells stimulated with the combination of IL-4/GM-CSF demonstrated CD23 expression along with changes in cell morphology including depolymerization of isoactin fibers, cell spreading, and membrane ruffling (Figure 4B). These changes in phenotype are consistent with flow cytometry results in that the larger cells expressed CD23 (Figure 4D). In contrast, the control BSA stimulated population showed no changes in cell cytoskeletal structure and morphology (Figure 4A) or specific staining for CD23 (Figure 4C).

To confirm activity of protein synthesis, the protein content of the control and the experimental groups of cells were compared using a BCA protein analysis kit. Human ASMC were starved for 24 hours in 0.1% FBS containing medium M199 and then stimulated with BSA (1 μg/mL), IL-4 (0.5 nM), GM-CSF (0.4 nM), or IL-4 (0.5 nM)/GM-CSF (0.4 nM) for 24 hours. The protein content was increased by 19% in the IL-4/GM-CSF treated cells above that of the control (Table 2). The increase in protein concentration with IL-4 alone was not statistically significant.

**Table 2:** IL-4/GM-CSF Combination Increases Protein Content in huASMC.

Cytokine (nM)	mg/10^6 ^cells (n = 3)
BSA (vehicle)	1.17 ± 0.08
IL-4 (0.5)	1.18 ± 0.01
GM-CSF	1.12 ± 0.03
IL-4 (0.5)/GM-CSF (0.4)	1.39 ± 0.02 *

Alpha-smooth muscle isoactin positive huASMC (Clonetics) in T-75 flasks were starved for 24 h in 0.1% FBS containing medium M199. The cells were then stimulated with BSA (1 μg/ml)), IL-4 (0.5 nM), GM-CSF (0.4 nM), or IL-4/GM-CSF (0.4/0.5 nM) for 24 h. The cell lysates in RIPA buffer were analyzed for protein content using a commercially available BCA kit (Pierce). The optical density was read using a Bio-Kinetics EL-312 Microplate reader. Results are mean ± SEM (n = 3). *denotes value significantly different from the BSA vehicle treated control.

### Stimulation of huASMC with IL-4 induces phosphorylation of STAT-6 and expression of IL-2Rγc

IL-4 binds the IL-4R with high affinity, and signaling through IL-4 causes enhanced expression of IL-4R [21]. The induction of these genes is mediated through signal transduction molecules including signal transducer activator of transcription (STAT-6). The binding of IL-4 to its receptor complex induces the formation of an IL-4 receptor complex which consists of IL-4Rα and the common gamma chain (γc) of the receptors for IL-2, IL-4, IL-7, IL-9, IL-15, and IL-21 [21]. It has not been previously reported that airway smooth muscle cells express the IL-2Rγc, the signaling unit of the IL-4 receptors.

**Figure 5:**
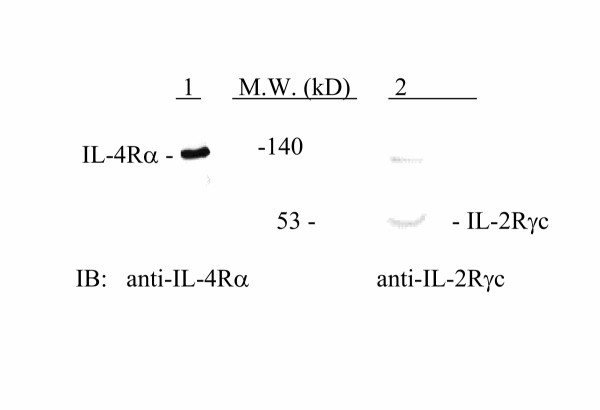
**IL-4Rα and IL-2Rγc Expression in huASMC**. A: IL-4Rα and IL-2Rγc expression in huASMC at baseline. Unstarved huASMC lysates were subjected to western blot analysis for IL-4Rα and IL-2Rγc using polyclonal anti-IL-4Rα (1:500 dilution, Santa Cruz), or monoclonal anti-IL-2Rγc (1:125 dilution, R&D Systems, Inc.). The nitrocellulose membranes were incubated with a 1:1,000 dilution of anti-rabbit or anti-mouse horseradish peroxidase linked whole antibody (Amersham). The immunoreactive protein bands were detected by ECL (Amersham). IL-2Rγc is minimally expressed in huASMC while IL-4Rα is expressed abundantly in huASMC.

**Figure 6:**
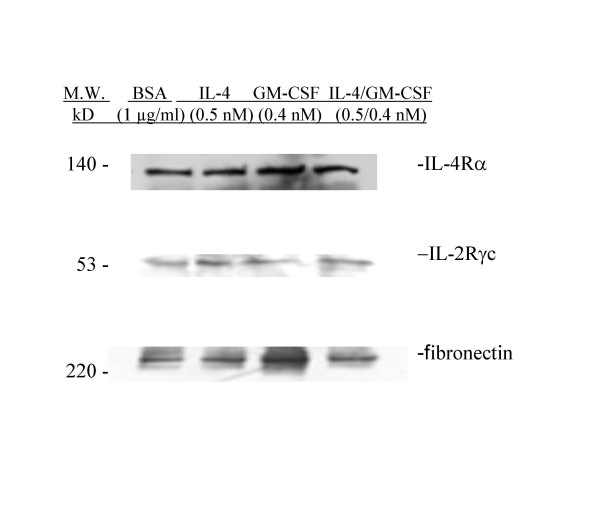
**Upregulation of IL-2Rγc Expression in huASMC by IL-4 and IL-4/GM-CSF**. Alpha-smooth muscle isoactin positive Human ASMC (Clonetics) in T-75 flasks were starved for 24 h in 0.1% FBS containing medium M199. The cells were then either stimulated with BSA (vehicle) (1 μg/ml), IL-4 (0.5 nM), GM-CSF (0.4 nM), or IL-4/GM-CSF (0.5 nM/0.4 nM) for 24 hours. The IL-4 and IL-4/GM-CSF stimulated cells had increased IL-2Rγc expression compared to the BSA (vehicle) group. Fibronectin polyclonal rabbit antibody (Sigma) (1:250) was used as an irrelevant isotype control.

Western blot analysis of IL-4Rα and IL-2Rγc in huASMC lysates showed the presence of these receptor components on huASMC. Figure 5 shows abundant expression of IL-4Rα and a low level expression of IL-2Rγc protein on huASMC. After stimulation of huASMC with IL-4 (0.4 nM) for 24 h, a two fold increase in γc expression was observed compared to the BSA vehicle control (Figure 6).

**Figure 7:**
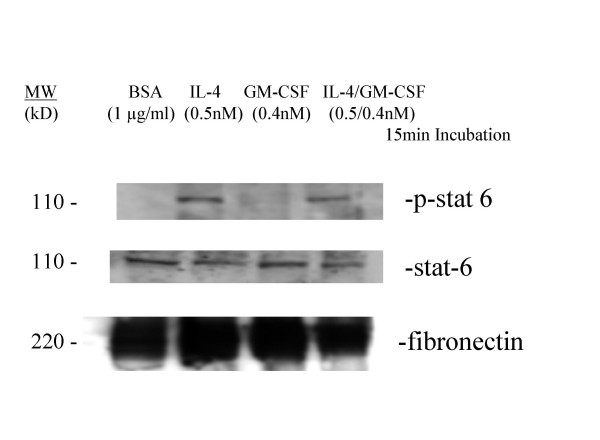
**Phosphorylation of STAT-6 by IL-4 and IL-4/GM-CSF in huASMC**. Starved huASMC were incubated with either BSA vehicle control (1 μg/ml), IL-4 (0.5 nM), GM-CSF (0.4 nM), or IL-4/GM-CSF (0.5 nM/0.4 nM) for 15 minutes. Standard Western blot analyses were performed to detect STAT-6 and phosphorylated-STAT-6 (p-STAT-6) using a anti-STAT-6 polyclonal rabbit antibody (Calbiochem) and anti-p-STAT-6 polyclonal rabbit antibody (Calbiochem). Anti-rabbit horseradish peroxidase linked antibody was used as the secondary antibody. Protein bands were detected by ECL. STAT-6 was abundantly expressed by all four groups, while p-STAT-6 was only expressed in the IL-4 and IL-4/GM-CSF groups. Fibronectin polyclonal rabbit antibody (Sigma) was used as an irrelevant isotype control and was abundantly expressed in all four groups.

To confirm that IL-4 was activating the IL-4Rα during the stimulation of huASMC, we examined the phosphorylation of downstream STAT-6 by western blot. Human ASMC were starved for 24 h and then stimulated with IL-4 (0.4 nM) for fifteen minutes. Results of Western blot revealed an approximately a four fold increase in intensity of the band for phosphorylated-STAT-6 in IL-4 stimulated cells when compared to BSA control (Figure 7). This supports the role of an IL-4 mediated signal transduction pathway involvement in CD23 upregulation in huASMC.

## Discussion

In our study, we have demonstrated that CD23, the low affinity IgE receptor, is upregulated on human airway smooth muscle cells by the cytokines IL-4, GM-CSF, and the combination of IL-4/GM-CSF. This upregulation of CD23 by the combination of IL-4 and GM-CSF was accompanied by an increase in cell volume and protein content, cytoskeletal depolymerization, cell spreading and membrane ruffling. Because ASMC require a doubling time of 48 hours, the increase in protein content could not be attributed to an increase in cell number. Also, the increase in cell number in gate D seen with LTD4 was likely secondary to the effects of ethanol (Table 1). Stimulation of huASMC by IL-4 caused an activation of STAT-6 and an increase in γc expression. Collectively, our findings suggest that CD23 expression can be stimulated by IL-4 and GM-CSF cytokines independent of IgE in huASMC and the upregulation of CD23 may play a role in cell migration and hypertrophy.

Previous studies have demonstrated an increase in CD23 expression in alveolar monocytes after stimulation with IL-4 and GM-CSF [10]. In that study, the use of the individual mediators alone did not increase the CD23 levels to that of asthmatic patients suggesting a possible synergistic role between IL-4 and GM-CSF. Our findings are consistent with these in that the combination of IL-4 and GM-CSF was most effective in upregulating CD23 in huASMC. Not all TH2 cytokines are involved in this process; IL-5 (0.4 nM) and IL-13 (0.4 nM) had no effect on CD23 expression. Cysteinyl leukotriene LTD4 (10 μM), prostaglandin PGD2 (10 μM) and tryptase (30 nM) did not induce CD23 expression on huASMC [26].

We evaluated the effect of stimulation of huASMC with IL-4 on phosphorylation of STAT-6 via the IL-4R which would confirm the presence of the receptor in huASMC. STAT-6 is a critical mediator of IL-4 stimulated gene activation, and it is regulated by both tyrosine and serine kinases [27]. It has been shown in a mouse model that STAT-6 binds the CD23a murine promoter, and STAT -/- mice stimulated with IL-4 are unable to upregulate CD23. This suggests STAT-6 is a critical mediator for IL-4 induced upregulation of CD23 [28]. IL-4 along with CD40 mediated signals are responsible for upregulation of CD23 on B cells [14]. In this study, we have confirmed the expression of the IL-4Rα and a low level of common gamma chain in huASMC and that after stimulation of huASMC with IL-4, there was a two fold increase in γc chain expression (Figure 6). Phosphorylation of STAT-6 after stimulation with IL-4 for 15 min confirms that IL-4 has bound and activated the IL-4 receptor complex (Figure 7). It has been shown that the common gamma chain is a functional β chain of the IL-4 receptor complex in certain cells [27], and our data suggest that this is the case in huASMC. Interestingly, IL-13 (4 nM, a concentration sufficient to simulate huASMC proliferation, unpublished observation) did not upregulate CD23. For proliferation of ASMC by IL-13, IL-4Rα and IL-13Rα1 are required for signal transduction and downstream activation of p44/42 extracellular regulated kinases (ERK, unpublished data). Apparently, IL-4Rα and IL-13Rα1 engagement is not sufficient for CD23 expression, further supporting the role of γc chain in CD23 expression by IL-4. The signs of signal transduction in response to IL-4, and the increase in protein content of the cell in response to IL-4 and GM-CSF combination (Table 2) represent activation of transcription and translation of CD23 in this case. Coupling of GM-CSF and its receptor complex is known to activate ERK that may have contributed to the synergistic effect of GM-CSF on CD23 expression.

CD23 expression was associated with changes in cell morphology including depolymerization of isoactin fibers, cell spreading, and membrane ruffling (Figure 4B &4D). Actin in ASMC is in a dynamic state and undergoes polymerization-depolymerization during the contraction-relaxation cycle [29,30]. Membrane ruffling and cell migration involve signaling pathways including PI3-kinase, Rac and other Rho family G protein members in a variety of cell types, including vascular smooth muscle cells. Rac has an essential role in cell migration and regulation of the actin cytoskeleton [31,32]. Moreover, ASMC are capable of switching their phenotypes from contractile to synthetic phenotype that is mediated by Rho kinases [32,33].

In summary, we have demonstrated that the low affinity IgE receptor can be induced on huASMC by specific cytokines including IL-4, GM-CSF, and the combination of IL-4/GM-CSF. The combination of IL-4/GM-CSF also induced morphologic changes in the ASMC that may contribute to the synthetic function or migration. In addition, IL-4 and IL-4/GM-CSF stimulation of huASMC increased the protein content of the cell, suggesting hypertrophy.

## Conclusion

T helper type 2 cytokines including IL-4 have major role in asthma pathogenesis. GM-CSF is a hemopoetic growth factor, mostly released by activated monocytes and T cells. Additional sources of GM-CSF include epithelium of asthmatic airways [34] and human airway smooth muscle cells [6,35]. Therefore, the effect of GM-CSF on CD23 expression can be both via paracrine and autocrine mechanisms. Previous studies by Hakonarson et al. [16,17] have shown that upregulation of the CD23 receptor has been associated with proasthmatic changes in agonist-mediated ASM constrictor and relaxant responsiveness. Our study suggests that CD23 expression is associated with elements of hypertrophy (i.e. an increase in cell volume and protein content), thus consistent with their findings. Inhaled corticosteroids, the mainstay in treatment of asthma, effectively reduce inflammation and remodeling of the epithelium and basement membrane. However, no agents have been proven effective in reducing smooth muscle mass in asthmatic patients. Recent study results on anti-CD23 therapy showed decrease in serum IgE. Further studies to intervene the upregulation of CD23 expression by cytokines IL-4 and GM-CSF may open a new avenue to target smooth muscle hypertrophy, an important element of severe asthma [2].

## Competing interests

The author(s) declare that they have no competative intrests.

## List of abbreviations

huASMC: Human airway smooth muscle cells

FceRI: High-affinity receptor for IgE

FceRII (CD23): Low-affinity receptor for IgE

AHR: Airway hyperreactivity

PGD2: Prostaglandin D2

LTD4: Leukotriene D4

PE: Phycoerythrin

FBS: Fetal bovine serum

BSA: Bovine serum albumin

PBS: Phosphate bufferred saline

BCA: Bicinchoninic acid

FITC: Fluorescein Isothiocyanate

TRITC: Tetramethyl Rhodamine Iso-Thiocyanate

RIPA: Radio-immunoprecipitation assay

ECL: Enhanced chemiluminescence light

Rα: Receptor alpha

γc: Common gamma chain

FACS: Fluorescent Activated Cell Sorter

STAT: Signal Transducer Activator of Transcription

TH2: T helper cell type 2

ERK: Extracellular regulated kinases

## Authors' contributions

JTB participated in designing experiments and performing CD23 analysis by flow cytometry.

RKG carried out flow cytometry, immunofluorescence studies, and drafted the manuscript.

HM carried out western blot analyses.

DBL supervised all aspects of the project.

All authors have read and approved the final manuscript.
